# Clinical Effectiveness of a Separated-Type Oral Appliance for Obstructive Sleep Apnea in Japanese Patients

**DOI:** 10.3390/dj14070458

**Published:** 2026-07-21

**Authors:** Kazutaka Ikeda, Ryo Kunimatsu, Shuzo Sakata, Katsuhito Sugai, Shintaro Ogashira, Kotaro Tanimoto

**Affiliations:** 1 Department of Orthodontics, Division of Oral Health and Development, Hiroshima University Hospital, 1-2-3 Kasumi, Minami-ku, Hiroshima 734-8553, Japan; 2 Department of Orthodontics and Craniofacial Developmental Biology, Graduate School of Biomedical and Health Sciences, Hiroshima University, 1-2-3 Kasumi, Minami-ku, Hiroshima 734-8553, Japan

**Keywords:** obstructive sleep apnea, oral appliance, mandibular advancement device, separated-type appliance, polysomnography, apnea–hypopnea index

## Abstract

**Background/Objectives**: Oral appliance (OA) therapy is an established treatment option for patients with mild-to-moderate obstructive sleep apnea (OSA). Although OAs of various designs have been developed, clinical evidence regarding the effectiveness of separated-type OAs in OSA remains limited. We evaluated the clinical outcomes of patients with OSA treated using a custom-made separated-type OA. **Methods**: This retrospective study included 47 Japanese patients with OSA who were diagnosed using polysomnography and referred for OA therapy between March 2015 and March 2025. Baseline demographic and clinical characteristics were obtained from medical records for all patients. Treatment effectiveness was analyzed in the 27 patients who underwent both baseline and follow-up polysomnography. The normality of within-patient Apnea–Hypopnea Index (AHI) differences was assessed using the Shapiro–Wilk test, and paired comparisons were performed using the paired *t*-test. **Results**: Follow-up polysomnography after OA therapy was performed in 27 patients (57.4%). In these patients, AHI significantly decreased from 17.4 ± 8.6 events/h at baseline to 8.2 ± 5.1 events/h after OA therapy (mean reduction, 9.23 events/h; 95% CI, 6.33–12.12; paired *t*-test, *p* < 0.001). OSA severity classification improved after treatment, and no patients were classified as having severe OSA at follow-up. **Conclusions**: Treatment with a custom-made separated-type OA was associated with a significant reduction in AHI among patients who underwent follow-up polysomnography, suggesting that separated-type OAs may be a useful option for selected patients with mild-to-moderate OSA. These findings should be interpreted with caution because of the retrospective design and incomplete follow-up data.

## 1. Introduction

Obstructive sleep apnea (OSA) is a disorder characterized by repeated obstruction or narrowing of the upper airway during sleep, leading to apnea and hypopnea. OSA is associated with an increased risk of cardiovascular diseases, such as hypertension, myocardial infarction, and stroke, and significantly affects the quality of life and overall health [1]. Continuous positive airway pressure (CPAP) therapy is the established first-line treatment for OSA, as it effectively prevents blood oxygen level decline during sleep, thereby ensuring high-quality sleep. However, reports of its effectiveness in preventing cardiovascular events are inconsistent, and low treatment adherence is a notable issue, particularly in patients with mild symptoms [2,3].

In adults with OSA, oral appliances (OAs) can improve anatomical airway narrowing by advancing the mandible, thereby improving sleep quality and reducing daytime sleepiness. Some studies have reported that the blood pressure-lowering effect of OAs is comparable to that of CPAP, particularly in cases of mild-to-moderate OSA or in patients for whom CPAP is unsuitable [4]. In Japan, OA therapy is widely used in adults with mild-to-moderate OSA [5]. Various types of OAs, such as monobloc, separated-type, and tongue-retaining devices, are available [6]. The monobloc and separated-type devices expand the airway to improve apnea by advancing the mandible and are effective in many patients [7,8]. However, discomfort caused by the device and changes in occlusion can lead to treatment discontinuation, highlighting the importance of long-term follow-up and management of side effects [9].

Separated-type OAs include independent components for the upper and lower jaws, allowing partial mandibular movement during device use. Compared with the monobloc type, which is associated with a sense of restriction, allowing partial mandibular movement may reduce the psychological burden associated with separated-type OA use, as evidenced by favorable subjective evaluations of sleep quality [9]. Nevertheless, owing to the cost and complexity of fabrication, clinical adoption of these devices is limited, and reports on the treatment outcomes of separated-type OA in Japan are scarce. In this study, we conducted a retrospective study to evaluate the clinical effectiveness of a separated-type OA custom-made in our department for treating patients with OSA.

## 2. Materials and Methods

Forty-seven patients diagnosed with OSA by a medical specialist using polysomnography (PSG) who were deemed suitable for OA treatment were referred to the Orthodontic Department of Hiroshima University Hospital between March 2015 and March 2025.

Eligibility for OA therapy was determined jointly by a sleep physician and an orthodontist. Patients were considered suitable when OSA had been confirmed by PSG and they met one of the following criteria: mild-to-moderate disease (5 ≤ AHI < 30), or severe disease (AHI ≥ 30) with intolerance of, or refusal of, CPAP. Additional requirements included a sufficient number of sound teeth to retain the appliance and an adequate mandibular protrusive range. Patients with active periodontal disease, untreated caries, temporomandibular joint disorders, or central sleep apnea were not considered candidates for OA therapy.

This study was approved by the Ethics Committee of Hiroshima University (Approval No. E2015-0056-04; date of approval: 31 March 2025). All procedures performed in this study were in accordance with the ethical standards of the Ethics Committee of Hiroshima University and with the 1964 Helsinki Declaration and its later amendments or comparable ethical standards. Given the retrospective nature of the study, the requirement for written informed consent was waived by the Ethics Committee. An opt-out procedure was applied, and study information was disclosed on the institutional website (https://med.ethics-system.hiroshima-u.ac.jp/rinri/publish.aspx) (accessed on 16 July 2026).

From the patients’ medical records, we retrospectively obtained data on age, sex, body mass index (BMI), the Apnea–Hypopnea Index (AHI) at the initial PSG, AHI at the follow-up PSG after OA fitting, and whether patients continued regular visits. The decision to conduct follow-up PSG was based on patients’ continued visits and the medical specialist’s judgment.

**Figure 1:**
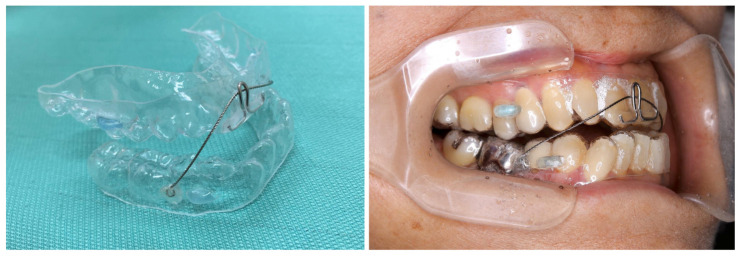
Structure of the separated-type oral appliance. The upper and lower jaws are designed as independent structures, with a wire attached to the lower jaw that can be hooked onto a hook placed on the upper anterior teeth to adjust the amount of forward movement. This design permits forward movement while maintaining a certain degree of freedom for the lower jaw.

The OA used in this study was a separated-type device with independent upper and lower jaw components that are connected by hooking a wire attached to the mandibular device onto a hook set on the maxillary anterior teeth, allowing partial mandibular movement during OA wear (Figure 1). The maxillary and mandibular components of the OA were fabricated using acrylic resin. Impressions were obtained using standard dental impression materials, and bite registration was performed at an appropriate mandibular advancement position. The upper and lower appliances were connected using a metal wire system (diameter: 0.0195 inch), allowing for adjustable mandibular advancement while maintaining partial mobility. The initial mandibular advancement was set at approximately 50–75% of each patient’s maximum protrusive range, according to symptoms and tolerance. Patients were instructed to wear the appliance nightly during sleep.

Continuous variables are presented as mean ± SD, and categorical variables as *n* (%). The effectiveness analysis was restricted to the 27 patients who underwent both baseline and follow-up PSG, using each patient’s own paired values. Normality of the within-patient AHI differences was assessed with the Shapiro–Wilk test; as the differences were normally distributed (*p* = 0.17), baseline and follow-up AHI were compared using the paired *t*-test.

The change in AHI is reported with 95% confidence intervals and the effect size as Cohen’s dz. The change in OSA severity category within these 27 patients was assessed using the Wilcoxon signed-rank test. Treatment success was defined a priori as a follow-up AHI < 10 events/h with a ≥50% reduction from baseline. To explore potential attrition bias, baseline characteristics were compared between patients with and without follow-up PSG (*n* = 27 vs. 20): age using the independent *t*-test, baseline AHI using the Mann–Whitney U test, and sex using Fisher’s exact test. Because this was a retrospective single-center study, no a priori sample size calculation was performed. A sensitivity power analysis was performed using G*Power 3.1.9.7 for the paired comparison of AHI in patients with both baseline and follow-up PSG data. With 27 paired observations, a two-tailed α level of 0.05, and a power of 0.80, the minimum detectable effect size was Cohen’s dz = 0.56. A two-sided *p* < 0.05 was considered significant. Analyses were performed with Bellcurve for Excel version 4.10 (Social Survey Research Information Co., Ltd., Tokyo, Japan).

## 3. Results

The mean age of the patients was 53.2 ± 15.1 years, of whom 30 were men (64%) and 17 were women (36%). Most patients were in their 50 s to 60 s. The average AHI at the initial consultation was 17.1 ± 8.6 events/h, with similar mean AHI values in male and female patients (18.1 ± 9.7 events/h vs. 15.3 ± 5.2 events/h). OSA severity was classified as follows: mild when 5 ≤ AHI < 15, moderate when 15 ≤ AHI < 30, and severe when AHI ≥ 30 [10]. At the initial consultation, the OSA severity classification was mild in 46.8%, moderate in 44.8%, and severe in 8.4% of cases (all of whom were intolerant to CPAP).

**Figure 2:**
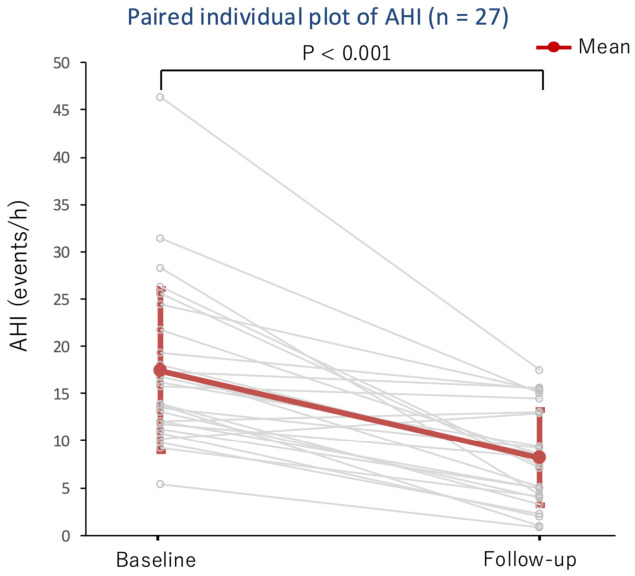
Individual changes in the Apnea–Hypopnea Index (AHI) before and after treatment with the separated-type oral appliance in the 27 patients who underwent follow-up polysomnography. Each gray line represents one patient, connecting baseline and follow-up AHI values. The red line indicates the mean value. AHI significantly decreased after treatment (paired *t*-test, *p* < 0.001).

Follow-up PSG was performed in 27 of 47 cases (57.4%). Among these 27 patients, the AHI decreased significantly from 17.4 ± 8.6 events/h at baseline to 8.2 ± 5.1 events/h after OA therapy (paired *t*-test; mean reduction 9.23 events/h, 95% CI 6.33–12.12; *p* < 0.001) (Table 1, Figure 2). The 27 patients who underwent follow-up PSG comprised 15 men and 12 women, with a mean age of 56.3 ± 13.1 years and a mean baseline AHI of 17.4 ± 8.6 events/h (Table 2). Within these 27 patients, the OSA severity distribution shifted from mild 48.1%, moderate 44.4%, and severe 7.5% at baseline to normal 37.0%, mild 44.4%, and moderate 18.5% after treatment, with no patient classified as severe (Wilcoxon signed-rank test, *p* < 0.001). Using a predefined criterion of a follow-up AHI < 10 events/h with a ≥50% reduction, treatment was successful in 15 of 27 patients (55.6%). Among the 47 patients, 16 (34%) continued long-term regular visits after OA fitting.

To assess potential attrition bias, baseline characteristics were compared between patients who did and did not undergo follow-up PSG (Table 3). Age, sex distribution, and baseline AHI did not differ significantly between patients with and without follow-up PSG (*p* = 0.117, *p* = 0.226, and *p* = 0.768, respectively). However, residual attrition bias cannot be excluded.

## 4. Discussion

In this study, treatment with a custom-made separated-type OA was associated with a significant reduction in AHI among patients who underwent follow-up PSG. These findings suggest that a separated-type OA may be a useful treatment option for selected patients with mild to moderate OSA.

Our findings are also consistent with the mechanism by which separated-type OAs advance the mandible to prevent posterior movement of the tongue base and promote opening of the upper airway. Gao et al. [11] and Jugé et al. [12] have reported a strong correlation between the amount of mandibular advancement and enlargement of the pharyngeal cross-sectional area through magnetic resonance imaging (MRI)-based analysis of upper-airway morphological changes. Previous studies have reported significant improvements in upper-airway morphology with the use of OAs. Specifically, enlargement of the pharyngeal cavity diameter or cross-sectional area at the soft palate and tongue base has been consistently demonstrated in many earlier studies. Using lateral cephalometric analysis, Doff et al. demonstrated a significant increase in the posterior airway space at the uvula and tongue base after OA placement in patients with OSA [13]. Furthermore, using nasal endoscopy, Ryan et al. revealed a significant increase in the pharyngeal cross-sectional area of the hypopharyngeal region, including the posterior palate and tongue base [14]. Additionally, Sutherland et al. demonstrated an increase in the lateral and anteroposterior diameters of the soft palate and expansion of the pharyngeal cavity owing to anterior movement of the soft tissue surrounding the tongue base, providing three-dimensional evidence of the upper-airway expansion effect [15]. The improvement in AHI observed with the separated-type OA in the present study was likely achieved through expansion of the upper-airway diameter from the soft palate to the tongue base, consistent with these physiological mechanisms.

Although daytime sleepiness was not evaluated, it is clinically relevant when interpreting OSA improvement. Sleep-disordered breathing in adults, particularly those with OSA, is characterized by excessive daytime sleepiness (EDS) as a major clinical symptom. EDS is widely used as an indicator of the severity and clinical significance of OSA [16]. In addition to obesity, craniofacial morphological factors, such as mandibular retrusion and increased facial height, are also involved in the onset of adult OSA, which potentially causes sleep-disordered breathing through morphological narrowing of the upper airway. Malocclusion is a clinical feature reflecting craniofacial morphological disharmony and has been suggested to be a risk factor for sleep-disordered breathing in adults, based on EDS prevalence [17]. Therefore, the effectiveness of the OA device is considered to be related to anatomical structures.

Lee et al. compared the therapeutic effects of monobloc and separated-type devices, reporting that the monobloc was superior in terms of AHI improvement and response rate [18]. In a meta-analysis, Ishiyama et al. demonstrated that although monobloc appliances yielded greater AHI improvement, the two types of OAs did not differ significantly in terms of minimum SpO_2_, Epworth Sleepiness Scale scores, or adverse events [19]. Özköylü et al. reported that although AHI improvement was comparable between the two types of OAs, upper-airway volume expansion was slightly greater with monobloc devices [20]. In contrast, Lee et al. reported a higher long-term continuation rate with separated-type OAs, likely because the greater freedom of mandibular movement improved comfort during use [18]. Thus, although monobloc appliances may provide greater reductions in AHI, previous reports suggest that separated-type appliances may offer advantages in comfort and adjustability; these subjective aspects were not assessed in the present study. Previous studies suggest that patient characteristics and the amount of mandibular advancement may influence outcomes, although these relationships were not examined in the present study.

Further research regarding structural changes and the risk of damage to the device with long-term use is warranted. Although occlusal changes were not evaluated in the present study, they are a recognized consequence of long-term OA use and merit consideration. Doff et al. reported significant reductions in overjet and overbite and decreased molar contact over 2 years, related to the amount of mandibular advancement [21], and Ishida et al. observed comparable anterior-tooth inclination and molar changes over 4 years in Japanese patients [22]. A 5-year study suggested that such changes, although progressive, may stabilize as a new occlusal balance develops [23]. These occlusal changes are better regarded as predictable treatment effects than unexpected complications; adequate pre-treatment explanation, occlusal monitoring during use, and timely adjustment are therefore advisable, and prospective evaluation of occlusal and temporomandibular outcomes is warranted.

This study had several limitations. First, repeat PSG was conducted in approximately 57% of all cases, limiting the evaluation of treatment effects to only part of the study population. Although baseline age, sex distribution, and baseline AHI did not differ significantly between patients with and without follow-up PSG, residual selection or attrition bias cannot be excluded. Second, because no direct comparison with monobloc OA or CPAP was performed, we could not quantitatively demonstrate the specific effects of the separated-type OA relative to other treatments. Third, potentially relevant confounding factors, including BMI, weight change, craniofacial morphology, degree of mandibular advancement, subjective symptoms, objective adherence, and occlusal changes, were not consistently available from the retrospective medical records and therefore could not be accounted for in the analyses. Finally, because this was a retrospective single-center study, no a priori sample size calculation was performed. Although the sensitivity analysis indicated that the follow-up PSG subgroup had sufficient sensitivity to detect a moderate within-subject effect, the sample size remained limited for detecting small effects and for reliable subgroup analyses. These limitations indicate that the results should be interpreted carefully and confirmed in prospective controlled studies.

## 5. Conclusions

In this single-center retrospective study, treatment with a custom-made separated-type OA was associated with a significant reduction in AHI among the 27 Japanese patients with paired baseline and follow-up PSG data. These findings suggest that separated-type OAs may be a useful option for selected patients with mild to moderate OSA. Given the exploratory design, partial follow-up, and absence of a control group, these findings should be interpreted with caution and confirmed in prospective, adequately powered, multicenter studies with longer follow-up and imaging-based functional evaluation.

## Figures and Tables

**Table 1:** Paired comparison of the AHI before and after treatment in the 27 patients with follow-up PSG.

Group (*n*)	Baseline AHI	Follow-Up AHI	Mean Change (95% CI)	Reduction (%)	*p*
Overall (*n* = 27)	17.4 ± 8.6	8.2 ± 5.1	9.23 (6.33 to 12.12)	51.1	<0.001
Male (*n* = 15)	19.1 ± 10.0	8.6 ± 5.2	10.53 (5.78 to 15.29)	52.2	<0.001
Female (*n* = 12)	15.4 ± 6.2	7.8 ± 5.0	7.59 (4.26 to 10.93)	49.6	<0.001

Values are presented as mean ± standard deviation unless otherwise indicated. *p* values were calculated using the paired *t*-test. Reduction rate was calculated for each patient as (baseline AHI − follow-up AHI)/baseline AHI × 100, and the mean value is shown. AHI, Apnea–Hypopnea Index; CI, confidence interval; PSG, polysomnography.

**Table 2:** Demographic and clinical characteristics.

Item	All Patients (*n* = 47)	Follow-Up Cohort (*n* = 27)
Number of cases	47	27
Age (years), mean ± SD	53.2 ± 15.1	56.3 ± 13.1
Male/Female, *n* (%)	30 (64%)/17 (36%)	15 (55.6%)/12 (44.4%)
BMI (kg/m^2^), mean ± SD	23.2 ± 4.0	22.4 ± 3.8
Baseline AHI (events/h), mean ± SD	17.1 ± 8.6	17.4 ± 8.6
Severity, mild/moderate/severe	46.8%/44.8%/8.4%	48.1%/44.4%/7.5%

AHI, Apnea–Hypopnea Index; BMI, body mass index; SD, standard deviation.

**Table 3:** Baseline comparison of patients with and without follow-up PSG.

Variable	Follow-Up PSG (*n* = 27)	No Follow-Up PSG (*n* = 20)	*p*
Age (years), mean ± SD	56.3 ± 13.1	49.0 ± 16.9	0.117
Male/Female, *n*	15 (55.6%)/12 (44.4%)	15 (75%)/5 (25%)	0.226
Baseline AHI (events/h), mean ± SD	17.4 ± 8.6	16.7 ± 8.7	0.768

AHI, Apnea–Hypopnea Index; PSG, polysomnography; SD, standard deviation.

## Data Availability

The raw data supporting the conclusions of this article will be made available by the authors on request.

## Abbreviations

The following abbreviations are used in this manuscript:

AHI	Apnea–Hypopnea Index
CPAP	continuous positive airway pressure
OA	oral appliance
OSA	obstructive sleep apnea
PSG	polysomnography
MRI	magnetic resonance imaging
EDS	excessive daytime sleepiness
